# Supplementary figures and images for: Task dynamics define the contextual emergence of human corralling behaviors
Source: PLoS One. 2021 Nov 15;16(11):e0260046. doi: 10.1371/journal.pone.0260046 (PMC8592491; doi:10.1371/journal.pone.0260046)

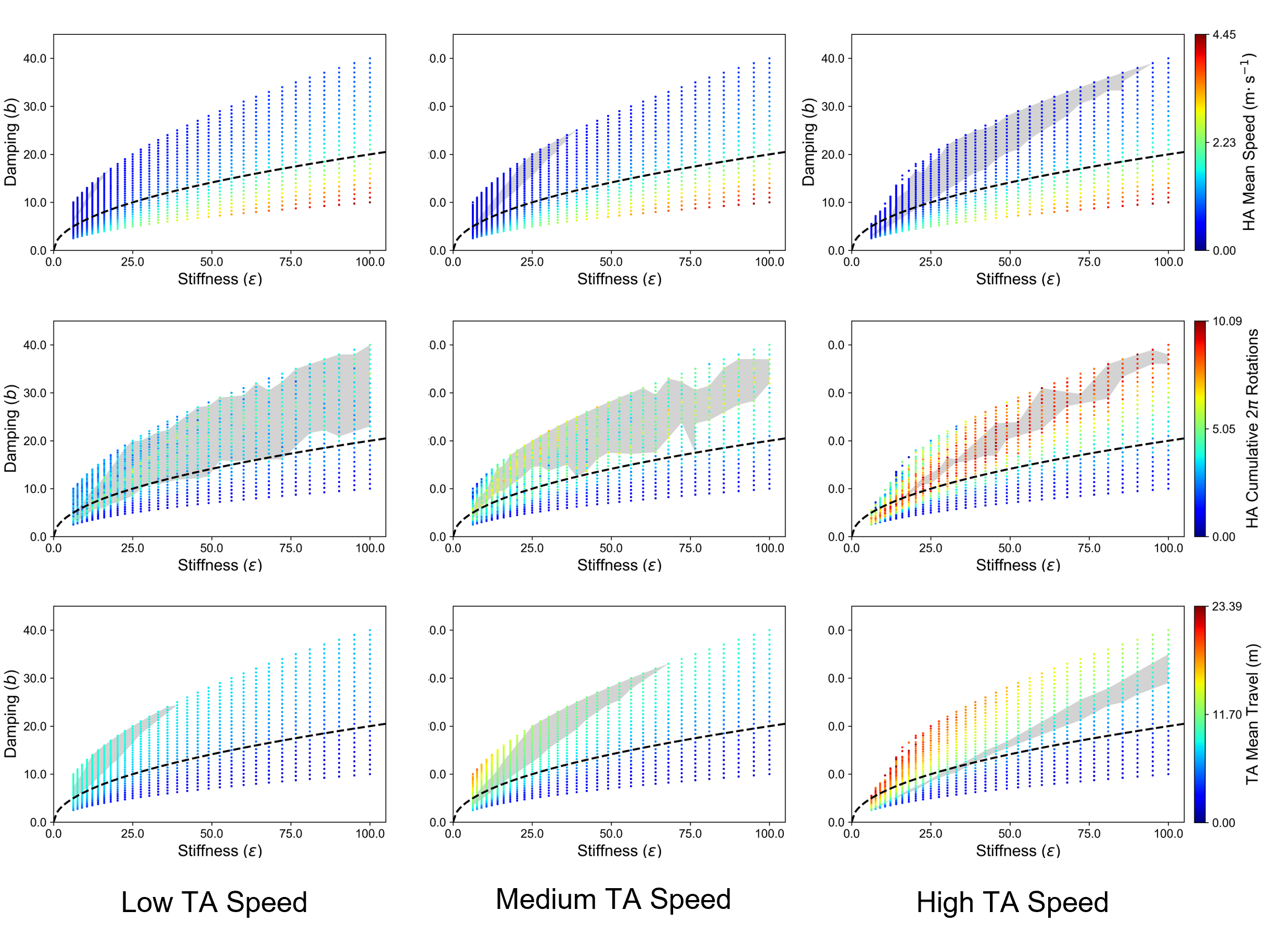

Supplement: S1 Fig — The gray areas in the modified figure represent the 95% CI of human mean locomotion speed (HA Mean Speed, top row), human Cumulative 2π Rotations (middle row) and TA Mean Speed during the human experiment (bottom row). The gray areas are displayed separately for the low- (left column), medium- (middle column) and high-speed conditions (right column). (TIF) [file pone.0260046.s001.tif]
