# Supplementary material for: Task dynamics define the contextual emergence of human corralling behaviors
Source: PLoS One. 2021 Nov 15;16(11):e0260046. doi: 10.1371/journal.pone.0260046 (PMC8592491; doi:10.1371/journal.pone.0260046)
Supplement: Attachment — Submitted filename: NalepkaEtAl2021_ResponseToReviews_Final.pdf [file pone.0260046.s008.pdf]

Response to Reviews for Submission PONE-D-21-23404

RE: *"Task dynamics define the contextual emergence of human corralling behaviors"* submitted for publication with *PLOS ONE*

Dear Dr. Bryan C. Daniels,

We would like to thank you and both reviewers for their positive and constructive feedback on our manuscript. We have modified the document to address the comments raised by the reviewers and believe the newly improved document now meets the high standards of *PLOS ONE* and believe it is acceptable for publication.

For your convenience, we have included excerpts of the editorial office and reviewers' comments in this document in **blue text**, with our responses in **black text**.

Comments from the Editorial Office:
Please ensure that your manuscript meets PLOS ONE's style requirements, including those for file naming.

The revised manuscript and included files now meet *PLOS ONE*'s style requirements.

Please remove any funding-related text from the manuscript and let us know how you would like to update your Funding Statement.

Funding information has now been removed from the Acknowledgement section. The Funding Statement should read as follows, as suggested by the Editorial Office:

PN was supported by the Macquarie University Research Fellowship. AC was supported by the Charles Phelps Taft Research Center at the University of Cincinnati. MJR was supported by the Australian Research Council Future Fellowship (https://www.arc.gov.au/) (FT180100447). RWK, ES and MJR were supported by the National Institutes of Health (https://www.nih.gov/) (R01GM105045). The funders had no role in study design, data collection and analysis, decision to publish, or preparation of the manuscript.

Comments from Reviewer 1:
The paper is well written and attempts to make a connection between human behavior in encirclement experiments in humans and a mathematical model that is supposed to explain this behavior.

Thank you for your comment and summary of the goal for the manuscript.

However, this connection is presented in Fig.6 and it is unclear:

- As explained in the text and Fig. 5 there are two qualitatively different behaviors that appear in the simulations: circling movements (overdamped) and oscillatory behavior (underdamped). If the gray areas in Fig. 6 represent the experiments, it seems that most of the cases are in the overdamped region, so where is the experimental justification for the existence of both behaviors?

The gray areas in Fig. 6 represent the 95% CI of the movement behaviors of human participants who completed the virtual reality (VR) corralling task while locomoting (the task displayed in Fig. 3 and described in *Method*). Although we do not present new experimental data of participants exhibiting oscillatory behavior, this discovered behavior has been documented in a number of previous studies (Nalepka et al., 2017; Nalepka et al., 2019; Rigoli et al., 2020) where participants completed the task using hand movements. The findings of these previous studies are reviewed in the *Corralling behaviors in humans* section of the *Introduction* and are described in Fig. 1. Further, in the subsection titled *Model simulations reveal emergent dynamics that represents the strategies humans adopt in different corralling task contexts* in *Results*, we relate the oscillatory and circling behaviors of the artificial agents to the human locomotion dataset (for circling behavior), as well as our results from previous studies (for oscillatory behavior).

The observation of either oscillatory or circling behavior is dependent on how the stiffness and damping terms are parameterized. Parameters that correspond to over-/under-damped dynamics can be understood as reflecting the constraints acting upon human participants (as discussed on Lines 276 to 289 in *Results*). Compared to locomotion, hand movements across the task environment (like the one presented in Fig. 1) are less constrained which allows oscillatory behavior to be practically possible. As discussed in lines 302-309 in *Results*, participants in the locomotion-based study would be required to produce locomotion speeds that average the speeds required for running. As this would be difficult to maintain over the course of a 2-minute trial, participants select slower movement velocities which result in the emergence of coordinated circling behavior.

- The results of the simulations and experiments are mapped using HA mean speed, HA cumulative 2Pi roations or Mean Travel? According to the caption of the figure only HA mean speed is considered (?). All the three could be used and it will be important to check if the experimental data fall in the same region of the Damping-Stiffness coordinates. Otherwise the connection between this model and the experimental data is not convincing enough and it is supposed to be one of the main pillars of this study.

In Fig. 6, simulation results are presented for HA Mean Speed (top row), HA Cumulative 2π Rotations (middle row) and TA Mean Travel (bottom row) as a function of the model's damping and stiffness parameters. The gray areas that overlay the figures represent the 95% CI of human mean speeds for the three difficulty conditions (left, middle and right columns). These gray areas were generated using the simulation plots for HA Mean Speed (top row) and then copied for the subsequent figure's rows for visualization. When the gray areas are overlaid in this way, one can visualize the range of parameters that result in movement dynamics in the 'ballpark' of human capability (i.e., resulted in movements in the range of speeds that a human could reproduce from the observed human experiment).

We selected the 95% CI of human HA Mean Speed for the gray areas since stiffness and damping directly relate to movement dynamics and believe it to be an atheoretical quantity that can be used to relate to the other dependent measures (whereas if HA Cumulative 2π Rotations or Frequency was chosen, that presupposes the existence of circling or oscillatory behavior). However, we have now included an additional Supplemental Figure (S6) that includes gray areas to represent the 95% CI of the human result for that associated measure. Specifically, the Supplemental Figure includes grey areas that represent the 95% CI for human Mean Speed (top row), human Cumulative 2π Rotations (middle row) and TA Mean Travel in the human experiment (bottom row). As can be seen in the Supplemental

Figure, both the Mean Speed and Cumulative 2π Rotations are in the overdamped regions of the stiffness-damping parameter space.

If only Eqs. 1, 2 and 11 are relevant to the model, they should appear together. Eqs. 3-4 which are not part of the model should appear only in the discussion (where they are relevant).

Eq. 11 is now defined in the *Task-dynamic models of corralling evasive agents* subsection of the *Introduction* (now referred to as Eq. 3). Eqs. 3-4 (now Eqs. 4-5) are retained as we believe it helps motivate the current study. As discussed in *Current study* (Lines 198-207), the transition from S&R to COC and circling behaviors correspond to graph-dynamic changes in the composition of equations that describe these behaviors (specifically, from point attractive to limit-cycle dynamics). We believe the inclusion of these equations helps the reader see these differences. Further, we believe the inclusion of Eqs. 4-5 help illustrate the idea that the discovery of COC and circling behaviors may be viewed as latent dynamics of the behaviors described by Eqs. 1-3.

Is there a dependence of the results (experiments and simulations) on the number of TAs? As I understand that only 7 TAs were considered.

We kept the number of TAs to corral constant in this study to control for the attentional demands associated with tracking multiple objects. In our original experiment (Nalepka et al., 2017) we manipulated the number of TAs that participants had to corral (3, 5 or 7). Task difficulty was found to increase as a function of the number of TAs that need to be corralled. Additionally, the likelihood that participants discovered the coupled oscillatory containment (COC) strategy also increased as a function of the number of TAs to contain (this is discussed in the second paragraph of *Corralling behaviors in humans*, Lines 102-105). Further, related work which is currently under review demonstrate that the models described in this submission can allow for the successful containment of up to 60 TAs and has been evaluated with up to 4 HAs.

Comments from Reviewer 2:
Altogether, the paper reads as an excellent contribution to the literature. With highly impressive methods, and important conclusions. Specifically, the authors placed naive human dyads in a three dimensional virtual world using VR headsets, and asked them to corral a group of seven agents (I like to think of them as "prey"). They found that humans performed circling behaviours around the prey while keeping their partner as close to the opposite side of the circle as possible. The really critical component is that the authors were able to replicate some (although not all) aspects of this behaviour using similar task dynamic equations to their original set of papers.

Thank you for your comment and summary of the key takeaways for the submitted manuscript.

This suggests human minds may be running calculations similar to those suggested here. Does this suggest that early hunter gatherers were circling their prey? Or do we just have enough flexibility in our cognition to solve this task cognitively? The authors are, in my opinion, welcome to explore these questions or just ignore them.

Thank you for your comment. We cannot make any definitive statements about early human hunter-gatherers, but we find it remarkable the similitude in strategies observed by animals and other multi-agent systems when engaged in collaborative hunting, which was the inspiration for this manuscript. In our studies, human participants can discover coordinated oscillatory and circling behaviors, but their discovery is not guaranteed (second and third paragraph in the *Corralling behaviors in humans* section). As demonstrated by our simulations, artificial agents embodying a minimal task-dynamic model (Eqs. 1 and 2) can unveil these human collaborative behaviors as emergent properties. As discussed in the third paragraph of *Discussion*, what differentiates human discoverers from non-discoverers may either be that discoverers produce interactive behaviors that unveil these latent coordinative strategies, or that non-discoverers are not able to perceive these latent features when they are generated.

I think a summary paragraph would benefit this paper (again at authors discretion). Further than that, I have no major or minor comments and believe the paper is ready for publication in its present form. It read like a published work, and I must commend the authors on their clarity.

We thank you again for feedback regarding the quality of the manuscript and suggestion for a summary paragraph. Besides the *Abstract*, the manuscript includes a paragraph discussing the manuscript's aim in the third paragraph (Lines 75-88), as well as in *Current study*. Additionally, subheadings are used in the *Results* to provide a summary of the findings. The first paragraph in *Discussion* also provides a summary of the research findings.

Sincerely,
Dr. Patrick Nalepka on behalf of all the authors
